# Facile Synthesis of Three-Dimensional ZnO Nanostructure: Realization of a Multifunctional Stable Superhydrophobic Surface

**DOI:** 10.1371/journal.pone.0029047

**Published:** 2011-12-14

**Authors:** Jun Wu, Jun Xia, Wei Lei, Baoping Wang

**Affiliations:** School of Electronic Science and Engineering, Southeast University, Nanjing, People's Republic of China; University of Akron, United States of America

## Abstract

**Background:**

After comprehensive study of various superhydrophobic phenomena in nature, it is no longer a puzzle for researchers to realize such fetching surfaces. However, the different types of artificial surfaces may get wetted and lose its water repellence if there exist defects or the liquid is under pressure. With respect to the industry applications, in which the resistance of wetting transition is critical important, new nanostructure satisfied a certain geometric criterion should be designed to hold a stable gas film at the base area to avoid the wet transition.

**Methodology:**

A thermal deposition method was utilized to produce a thin ZnO seeds membrane on the aluminum foil. And then a chemical self-assemble technology was developed in present work to fabricate three-dimensional (3D) hierarchical dune-like ZnO architecture based on the prepared seeds membrane.

**Results:**

Hierarchical ZnO with micro scale dune-like structure and core-sharing nanosheets was generated. The characterization results showed that there exist plenty of gaps and interfaces among the micro-dune and nanosheets, and thus the surface area was enlarged by such a unique morphology. Benefited from this unique 3D ZnO hierarchical nanostructure, the obtained surface exhibited stable water repellency after modification with Teflon, and furthermore, based on solid theory analysis, such 3D ZnO nanostructure would exhibit excellent sensing performance.

## Introduction

It is well known that a lot of biomaterials, lotus leaves for example, possess an interesting capacity to maintain their surface clean. This unique property benefits from the presence of a hydrophobic wax layer covering over their micro- and nanostructured surfaces. The contaminants on the surface are easily removed as water droplet roll over the contaminated positions [1]. Inspired by this unique characteristics, superhydrophobic materials have attracted considerable attention and have been approved to possess the potential in applications ranging from self cleaning, anti-snow and resistance reduction to microfluidics [2]–[11]. Although this abnormal wetting behavior can be obtained from many micro- and nanofabrication methods, e.g. photolithography, electrodeposition, plasma etching, colloidal assembly, chemical vapor deposition, and templating et al [12]–[17], the superhydrophobic surfaces may get wetted and lose its water repellence if there are defects on the surfaces or the liquid is under pressure, which seriously restricted their practical applications.

In another case, a number of ZnO micro- and nanostructures possessing different diameters ranging from several nanometers to micrometers can be assembled evenly on a macro area [17]–[18]. As a result, the diversity in the morphologies has provided us a convenient ways to study the influence of micro- and nanostructures on the superhydrophobicity, and the non-defectively distributed nanostructure over the whole substrate provide us a superior candidate for the robust water resistant surface. Furthermore, the advantages and potential applications of ZnO micro- and nanostructures also opened the door for the realization of multifunctional superhydrophobic materials [19]. Particularly, in recent years, three dimensional ZnO nanostructures with complex morphology have received great research interest due to the fact that their advanced geometric structure and atom arrangement on the specific facets of these nanostructures can offer novel properties [20].

In present contribution, we introduced a facile chemical self-assemble technology to fabricate 3D hierarchical dune-like ZnO biomimic architectures, which is composed with core-sharing nanosheets on aluminum substrate. The obtained surface not only exhibited outstanding stability in water repellency comparing with our previously reported work introducing the high CA (contact angle) on ZnO surface [17], but also provided a deeper level of versatility, for example the large gaps space in the synthesized nanostructure is believed more advantageous for gas sensors, lithium-ion battery, catalysts and photocatalysis compared with those ordinary one- or two-dimensional nanostructures, because of their large surface area and facile mass transport in materials.

## Results and Discussion

**Figure 1:**
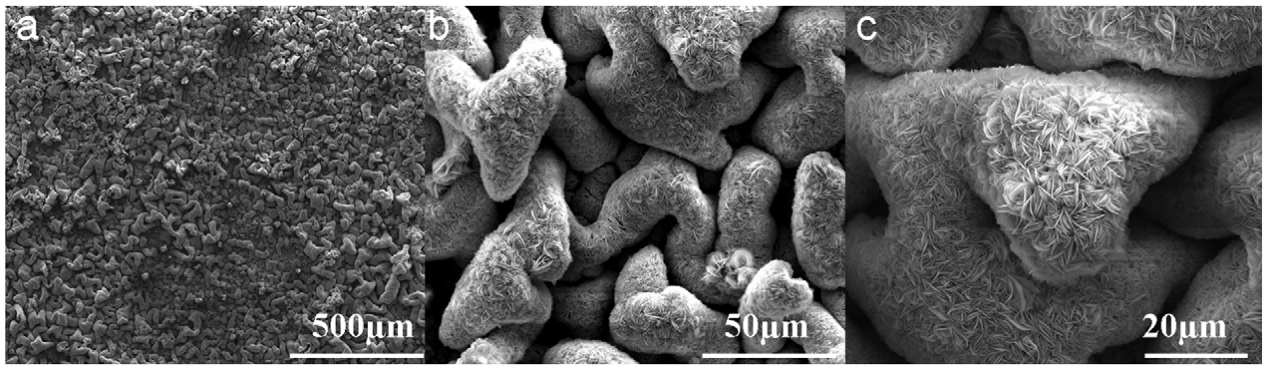
FE-SEM images of the ZnO film constructed on alumina substrate. The magnifications of these images are (a)×160 (b)×1600 (c)×3000 respectively.

**Figure 2:**
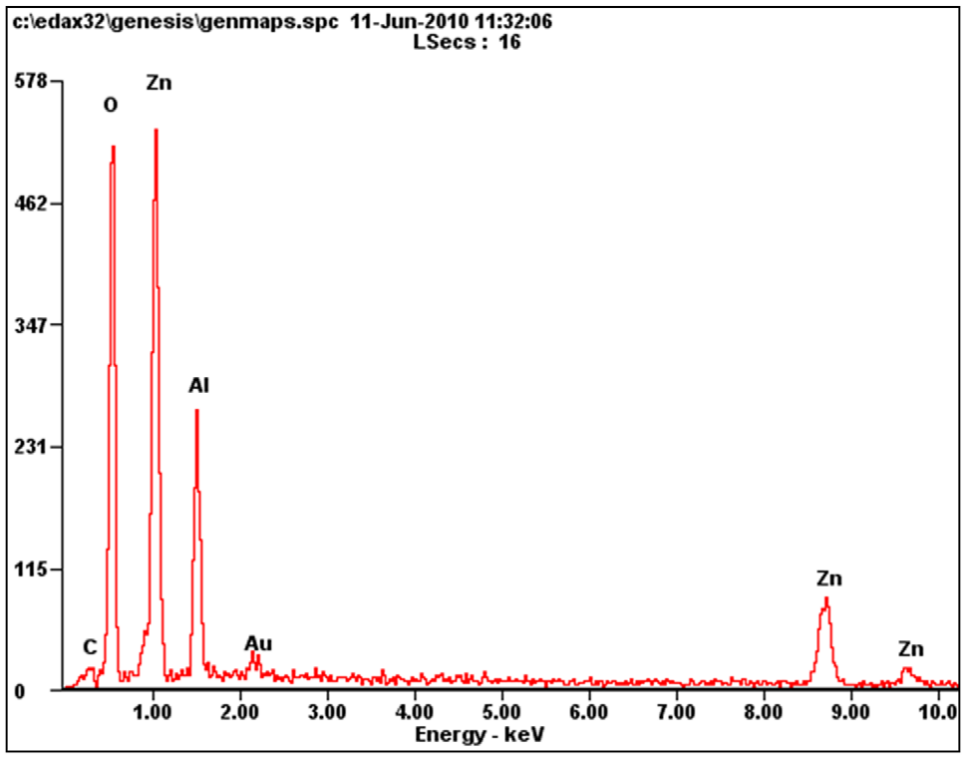
The energy dispersive X-ray (EDX) spectra of the sample.

FESEM images of the obtained film have been presented in Fig. 1, which indicate that the film is structured by evenly distributed dune-like architecture. By close observation of Fig. 1(b), it can be seen that these micro scale dunes are assembled by a large amount of core-sharing nanosheets. Plenty of gaps and interfaces are presented among these micro-dune and nanosheets, which increase the surface area of the materials and are potentially useful for applications such as wetting enhancement and sensor materials. To check the chemical composition of the product, we also recorded the energy dispersive X-ray (EDX) spectra as indicated in Fig. 2. These spectra indicated the presence of aluminum (substrate), together with expected zinc and large amount of oxygen, which means the hierarchical nanostructure, is composed with some kind of zincous oxide, e.g. ZnO.

It is well known that the driving force for the synthesis of nanocrystal is the decrease in Gibbs free energy at low supersaturation, and the morphology of the synthesized product is largely dependent on synthetic parameters such as reaction temperature, concentration, time, PH value and so on. Zhao J et al experimentally demonstrated that the zinc ion concentration strongly affected the growth rate and rod length, and had a smaller effect on rod diameter; the growth rate along [001] direction is more sensitive to temperature compared to those along [101] and [100] direction. The obtained nanocrystal would have a relatively greater length in [001] direction as the reaction temperature increasing from 30°C o 90°C [21].

Except from the above mentioned synthetic parameters control, the work reported herein has also utilized the substrate and seed-layer assisted approach to optimize the synthetic course. It is believed that the adopted aluminum substrate has acted a critical role in the formation of unique seed layer morphology, and then the high quantity and high interface energy of the seed interface on seed layer is supposed to play an important role in lowering the system energy and further facilitating crystal synthesis. In detail, we believed that the formation mechanism of the obtained distinctive morphology is benefited from following three factors. (1) The adopted substrate is aluminum foil, which has a distinct lattice constant compared with commonly used silicon, ITO and other kinds of substrates. In the ZnO seed layer deposition course, the lattice mismatch happened between the aluminum surface and the initial ZnO ultrathin membrane, which induced an accumulated strain force in the ZnO membrane. After the membrane thickness reached to a critical value, the dislocations happened to relax the strain force in the membrane, which might give birth to a unique pattern on the seed layer, and partially contributed to the distinctive hierarchical dune-like ZnO architecture. (2) For a seed layer assistant approach, to avoid mud crack in the gel membrane, the crosslinking agent is normally added into the precursor to combine the gel network. However, there is no such kind of additive in our deposition solution, which is supposed to be an important contribution for the formation a peculiar seed layer. (3) PH value has a crucial influence on the formation of ZnO nanosheets, the increased pH value in our experiment has weaken growth speed along the c-axis orientation, and thus the nanostructure transforms from ZnO nanorods to ZnO nanosheets.

**Figure 3:**
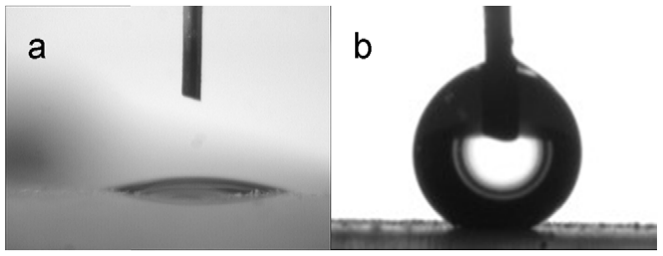
Droplet shapes on the prepared samples. Image (a) indicates the case before Teflon treatment and (b) indicates the case after Teflon treatment.

**Figure 4:**
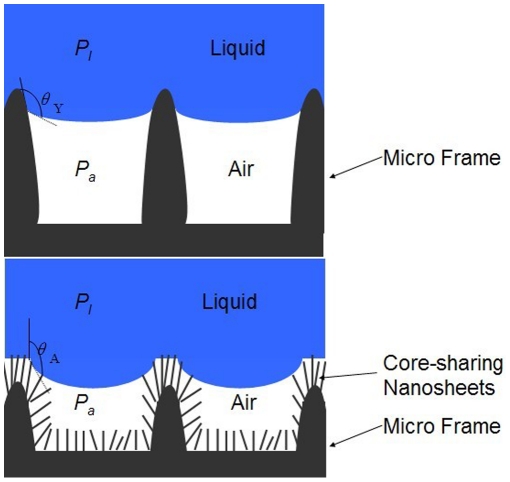
Schematic representation of the superhydrophobic states. Image (a) indicates the case on single micro scale frame and (b) presents the state on our 3D hierarchical dune-like ZnO architectures.

The obtained 3D hierarchical dune-like ZnO architectures demonstrated good hydrophilicity with water droplet contact angle less than 10°, and exhibited water repellent with water droplet contact angle larger than 160° after being deposited with a thin Teflon film by a spin coating approach. The distinct droplet shapes before and after Teflon treatment on the prepared sample are indicated in Fig. 3. This superhydrophobic behavior was attributed to the combination of the low surface energy coating and the micro or nano structures, and was repeated reported by the researchers in this field [3], [5]. We also introduced a high water CA surface based on a coralloid nanostructure in our previously reported work [17]. However, what we are concerning about in present work is some new nanostructures not only possessing a high water droplet CA, but also satisfying a certain geometric criterion which are capable to hold a stable gas film at the base area to avoid the wet transition. When these 3D hierarchical dune-like ZnO architectures were exposed to water droplet or even impacted with rain water, the water repellent property was still maintained with droplet sliding CA less than 5°. The difference of the contributions to the wettability between our 3D hierarchical dune-like ZnO architectures and the normal nanostructures is illustrated in Fig. 4. The additional roughness on the dune sidewall enlarges the gas fraction of the whole water/solid surface contact region. Based on this analysis, not only the water repellency is enhanced, the wetting transition to a wetting state is also delayed, which produced a more robust superhydrophobic biomimic surface. Actually, apart from the structural features introduced here, the feature size also played an important role in the wettability stability enhancement, which has been discussed detailedly by Lee [22].

In the research area of nano-scale sensors, many researchers consider the high surface-to-volume ratios as the main reason for the improved gas sensing performance. Nanomaterial with desirable morphology thus may help to improve their gas-sensing properties. As mentioned in previous section, plenty of gaps and interspaces among the micro-dunes and nanosheets increase the surface area of the synthesized crystal, which are therefore potentially useful for gas sensor applications. Actually as a family member of the semiconductor oxide gas sensors, ZnO gas sensors respond to the change of the carrier concentration, which is usually induced by oxygen adsorption on the surface of the sensing materials. When ZnO crystal adsorbed with negative oxygen ion, which is induced by electrons adsorption to conduction band of ZnO, exposed to reaction gas, the trapped electrons will back to the conduction band, and then the carrier concentration of ZnO will increase. Accordingly, the resistance of the sensor decreases. Zhang et al have reported sensor performances of different kind of ZnO nanostructures [23]. They observed that the 2 D hierarchically ZnO nanosheets performed enhanced sensitivity compared with ZnO powder. Furthermore, it is interesting to note that the 3D hierarchical ZnO superstructure possessed an even higher sensitivity compared with the 2D hierarchical ZnO nanosheets. They contributed the enhanced sensor sensitivity to the large surface area of the 3D hierarchical ZnO superstructure. Benefited from the reported 3D hierarchical dune-like ZnO architectures composited with core-sharing nanosheets, we believe that admirable sensing performance could achieve, which is considered as the main improvement compared with the previously reported superhydrophobic surfaces constructed with nanowires or micro scale frame which are only possessing limited surface area [17]–[18]. In another case, different kinds of ZnO morphology will induce totally different transference of the carriers and the electrons transport tunnel, which will further determine the performance of photovoltaic devices made of ZnO crystal. In this sense, the reported 3D ZnO biomimic architecture has provided a new opportunity of studying different kinds of ZnO nanostructure to optimize the design of photovoltaic devices.

In summary, the present study introduced a unique 3D ZnO hierarchical architecture by a facile chemical self-assemble technology. This cost-effective and simple fabrication method exhibites to be environmentally- and user-friendly, as no toxic reagent is needed. The 3D ZnO hierarchically architecture is demonstrated to be a satisfactory alternative to generate the artificial self cleaning, anti-snow and resistance reduction surface. Furthermore, the obtained surface is proved to possess the essential factors to fulfill the applications in gas sensors and photovoltaic devices.

## Methods

Chemicals such as Zn (CH_3_COO) _2_·2H_2_O, acetone, and ammonia were of analytical grade, and deionized water was used throughout the experiments. The detailed experimental process is described as follows. Firstly, an aluminum foil (with dimensions of 50×20×0.1 mm^3^) was pretreated by deionized water and acetone to remove surface oil contaminant, and was then used as the working substrate for the ZnO assemblage. The chemical self-assemble solution was prepared by dissolving certain amount of Zn (CH_3_COO) _2_·2H_2_O into deionized water together with ammonia on a sealable bottle, and forms a clear solution consists of 0.1 mol L^−1^ Zn (CH_3_COO) _2_·2H_2_O with PH value of 11. A thermal deposition method is utilized to introduce a thin ZnO seeds membrane. In detail, a deposition solution consists of 0.3 mol L^−1^ Zn (CH_3_COO) _2_·2H_2_O was sprayed onto the aluminium foil which was placing onto a hotplate with a working temperature of 280°C, and the spraying course was lasted for 30 minutes. After being cooled down to room temperature, the deposited aluminum foil was transferred into the prepared chemical self-assemble solution. Then, the bottle was sealed and heated to 90°C and subsequently kept at this temperature for 4 hours until the reaction was completed. After the bottle was air cooled down, the aluminum foil covered with the synthesized crystal film was taken out from the solution and washed with deionized water sufficiently, and successively got dried under vacuum at 80°C for 4 h before characterization. Morphology and structure characterization of the film was carried out by using field-emission Scanning Electron Microcopy and the component of the products was studied by Energy Dispersive X-ray Detector.
